# Computed microtomography visualization and quantification of mouse ischemic brain lesion by nonionic radio contrast agents

**DOI:** 10.3325/cmj.2013.54.3

**Published:** 2013-02

**Authors:** Marina Dobrivojević, Ivan Bohaček, Igor Erjavec, Dunja Gorup, Srećko Gajović

**Affiliations:** 1 Croatian Institute for Brain Research, University of Zagreb School of Medicine, Zagreb, Croatia; 2 Department of Anatomy, University of Zagreb School of Medicine, Zagreb, Croatia

## Abstract

**Aim:**

To explore the possibility of brain imaging by microcomputed tomography (microCT) using x-ray contrasting methods to visualize mouse brain ischemic lesions after middle cerebral artery occlusion (MCAO).

**Methods:**

Isolated brains were immersed in ionic or nonionic radio contrast agent (RCA) for 5 days and subsequently scanned using microCT scanner. To verify whether *ex-vivo* microCT brain images can be used to characterize ischemic lesions, they were compared to Nissl stained serial histological sections of the same brains. To verify if brains immersed in RCA may be used afterwards for other methods, subsequent immunofluorescent labeling with anti-NeuN was performed.

**Results:**

Nonionic RCA showed better gray to white matter contrast in the brain, and therefore was selected for further studies. MicroCT measurement of ischemic lesion size and cerebral edema significantly correlated with the values determined by Nissl staining (ischemic lesion size: *P*=0.0005; cerebral edema: *P*=0.0002). Brain immersion in nonionic RCA did not affect subsequent immunofluorescent analysis and NeuN immunoreactivity.

**Conclusion:**

MicroCT method was proven to be suitable for delineation of the ischemic lesion from the non-infarcted tissue, and quantification of lesion volume and cerebral edema.

Today, stroke is a major public health issue with substantial social and financial impact. For several decades, experiments with rodent cerebral ischemia have provided important insights into the mechanisms of stroke and subsequent brain repair. To induce ischemia in rodents, middle cerebral artery occlusion (MCAO) suture model is commonly used, because it corresponds to the ischemic stroke in humans (1,2). Cessation of glucose and oxygen supply leads to apoptotic and necrotic processes, which result in a breakdown of the blood-brain barrier and a consequent cerebral edema formation (3). Measuring the infarct volume and brain edema with different histological techniques, eg, triphenyltetrazolium chloride (TTC), hematoxylin-eosin, or Nissl, is widely accepted as suitable for animal ischemic stroke studies (4,5). Today, such histological techniques are easily accessible; however, they are two-dimensional (2D), labor intensive, time consuming, and maybe associated with significant tissue shrinkage and geometric distortions (6). As a possible alternative, magnetic resonance imaging (MRI) and computed tomography (CT), both nondestructive imaging methods, allow more precise, less time-consuming lesion reconstructions with advantages of three-dimensional (3D) analysis and a possibility to scan the same animals at multiple time points after cerebral ischemia.

MRI is an excellent tool not only for in vivo measuring of the evolution of ischemic lesion, but also for the study of fixed tissue specimens due to soft tissue contrast, nondestructive nature, and ability to provide 3D images. However, high field small-bore MRI (microMRI) scanners are highly expensive and require a significant effort of specially trained operators.

In contrast to this, we used x-ray computed tomography, the widely available diagnostic tool in emergency units, to explore the possibility of visualization of the mouse brain ischemic lesions. Today, the use of x-ray microcomputed tomography (microCT) technology enables noninvasive, high-resolution, in vivo and ex vivo imaging. MicroCT is effective for bone imaging (7,8), however soft tissue imaging requires the use of x-ray absorbing radiocontrast agents (RCA), due to small differences among the soft tissues in x-ray absorption (6). Although RCAs are routinely used in clinical radiography, only a few techniques are developed for imaging soft tissues of animal specimens: osmium staining of mouse embryos and honeybee brains, and reduced-silver nerve staining method for *Drosophila* brains (9-11). RCAs were shown to provide high-resolution microCT images of soft tissues, including the brain, with a contrast and spatial resolution comparable to microMRI scans (6,12). In addition, microCT scanners have the advantage of being substantially cheaper, and easier to maintain, thus being more accessible to research laboratories compared to MRI scanners.

The aim of this study was to explore if microCT scanning represented a valuable method for assessment of ischemic lesion size following transient cerebral ischemia in mice. Using Omnipaque RCA, microCT measurement of ischemic lesion size and cerebral edema significantly correlated with values determined by Nissl staining of serial brain sections. MicroCT quantification proved to be a method for evaluation of brain lesion size following ischemia.

## Materials and methods

All experiments were carried out on male inbred strain (C57Bl/6NCrl) mice (weight 22 ± 3 g). Age of the mice included in experiments was 2-4 months (young adults), as previously described (13,14). Ten non-operated animals were used to detect RCA with better gray/white matter contrast, as well as to detect optimal RCA dilution. Ten operated animals (8 stroked +2 sham operated) were used for the assessment of lesion size and brain edema with both, microCT and Nissl staining methods, and the values obtained by the two methods were compared. All animal procedures were approved by the University of Zagreb School of Medicine Ethics Committee and are in accordance with the Ethical Codex of Croatian Society for Laboratory Animal Science. All animals were allowed *ad libitum* access to water and food before and after surgery.

### Middle cerebral artery occlusion

As previously described (15,16), unilateral transient focal cerebral ischemia was induced by intraluminal filament occlusion of the middle cerebral artery during 1 hour, followed by reperfusion period of 24 hours. Briefly, animals (n = 10) were anesthetized with 2% isoflurane and surgical procedure was performed on heated surface in order to avoid hypothermia. One hour before and immediately after surgery, mice were injected with 1.0 mL 0.9% saline i.p. to prevent dehydration. After midline neck incision, left common, internal, and external carotid arteries were exposed. Silicon coated 6-0 monofilament (Doccol, Redlands, CA, USA) was inserted via external carotid artery into the internal carotid artery, and then into the circle of Willis, thus occluding middle cerebral artery. After an occlusion period of 60 minutes, the filament was removed, followed by 24-hour reperfusion period. Sham operated mice (n = 2) were operated identically as the MCAO, but the filament was incompletely inserted, in order to avoid occlusion of middle cerebral artery. Animals were monitored during surgery (heart rate and respiration), and treated with analgesics (buprenorphine [solution concentration 0.03 mg/mL, dosage 0.05-0.1 mg/kg] injections subcutaneously) right after surgery and after 12 hours in order to achieve postsurgical pain relief. Animals were able to eat and drink since food was softened with water, and water source was put low enough to be easily accessible. Animals were weighted before surgery and 24 hours after surgery.

### MicroCT contrast agents for the brain immersion protocol

The mice intended for imaging (n = 20) were anesthetized with intraperitoneal injection of 2.5% Avertin (Sigma Aldrich, St. Louis, MO, USA) and transcardially perfused with 30 mL of phosphate buffered saline (PBS), followed by 30 mL of PBS buffered 4% paraformaldehyde (PFA) (Sigma Aldrich). Brains were carefully dissected and postfixed overnight with 4% PFA at 4°C; the usual procedure for brain studies, as previously described (14).

Brains of non-operated animals (n = 10) were used for comparison of two different RCAs, in order to detect which one has better white/gray contrast. These brains were rinsed twice in PBS at room temperature and immersed in two different RCAs (n = 4/group): a low-osmolar, non-ionic iohexol monomer (Omnipaqe^®^, GE-HealthCare, Little Chalfont, UK) and a high-osmolar ionic contrast agent meglumine ioxithalamate monomer (Telebrix^®^, Guerbert, Roissy, France) for 5 days at room temperature, while two control brains were immersed in PBS. Series of different contrast dilutions 1:2 (final iodine concentration of 0.14 mol/dm^3^), 1:5 (0.28 mol/dm^3^), 1:10 (0.55 mol/dm^3^), and 1:20 (1.38 mol/dm^3^) diluted in PBS, were used for the experiment, as previously described (6,12).

According to the results obtained from the comparison of two different RCAs, the chosen nonionic RCA, Omnipaque, (RCA with better white/gray contrast) diluted 1:2 in PBS (optimal dilution to achieve best gray/white contrast) was used to compare brains of MCAO (n = 8) and sham operated (n = 2) mice. The brains were immersed in Omnipaque for 5 days at room temperature before microCT imaging.

### Micro CT imaging of brain ischemia

After immersion in different contrast agents, brains were scanned using SkyScan 1076 µCT scanner (Bruker, Kontich, Belgium). Before scanning, brains were blotted dry to remove any traces of RCA, wrapped in plastic foil, and placed into sample holder of the microCT device tube. In order to test whether there was dehydration, brains were weighted before and after the scanning and no difference was observed (data not shown). Probably, due to short scan time (15 minutes) and normal temperature inside the machine (26°C), the plastic foil prevented brain dehydration and shrinkage. Images were obtained using 48 kV and 200 µA, which corresponds to 18 µm spatial resolution, and 0.6° rotation step throughout 198°, which gives 331 projection images. Beam hardening effect of the x-rays was minimized by applying a 0.5 mm aluminum filter, to equalize the energy of the emitted x-rays from the source. Noise reduction on the images was accomplished by setting the frame averaging to a value of 5.

Data obtained by microCT device were further reconstructed by SkyScan NRecon (Bruker) software employing a range of 0-0.07 on a histogram scale, ring artifact reduction with a value of 5, and a 31% beam-hardening correction. Reconstructed images were aligned with SkyScan DataViewer software (Bruker) to identically position each brain with concurrent saving of transaxial data set. The left and right hemisphere of the brain were determined as the region of interest (ROI) and saved as a separate data set for further analysis within SkyScan CTAn software (Bruker).

In order to reveal which RCA showed better gray/white matter contrast and to detect optimal RCA's dilution, for calculation of standard x-ray attenuation units (Hounsfield units, HU) a phantom object was used. For this purpose, polypropylene tube of 1.5 mL volume was filled with deionized water and scanned in the microCT device using the same parameters used for brain scanning (6). To calculate the mean density value of HU for the water phantom, CTAn software was used. A calibrated value of 26.6846 HU was further used to measure HU in mouse brains stained with different RCA and in different dilutions. ROI consisting of 10 slices was analyzed in each white matter (corpus callosum) and gray matter (cortex), and HU were calculated and then plotted against I_2_ concentrations of the contrast agent.

Volumes of the lesion and hemispheres were manually traced with the same software, generated and calculated by interpolation across relevant slices within the reference range. High resolution 3D reconstruction imaging and analysis of the entire mouse brain with reconstruction of the ischemic lesion inside the ipsilateral infracted hemisphere was performed.

### Nissl staining

After microCT scanning, mouse brains were placed into 30% sucrose for dehydration and cryoprotection. After 3 days, brains were embedded in Tissue-Tek (O.C.T. compound, Sakura, Torrance, CA, USA), frozen-cut into 35 μm-thick coronal sections using cryostat, and stored at -20°C. Every sixth section was used for Nissl staining procedure. Sections were fixed in 100% methanol for 10 minutes, and further rehydrated in a series of decreasing alcohol baths (95% ethyl alcohol [EtOH] 15 min, 70% EtOH 2 min, and 50% EtOH 2 min), rinsed twice in distilled water for 2 min and stained with Cresyl Violet stain for 6 minž. After staining, sections were rinsed twice in water for 2 min and afterwards dehydrated in a series of increasing alcohol baths (50% EtOH 2 min, 70% acidic EtOH [1% glacial acetic acid in 70% EtOH] 2 min, 95% EtOH 2 min, 95% EtOH a few dips, and 100% EtOH 1 min). To lighten the sections, Histoclear (Invitrogen, Carlsbad, CA, USA) was applied for 5 minutes, after which slides were coverslipped with Histomount mounting media (Invitrogen). Slides were dried overnight and digitized using a Flatbed Epson perfection 4870 photo scanner (Epson America, Inc., Long Beach, CA, USA), with a resolution of 4800 dpi.

Images were analyzed using ImageJ 1.45 software (NIH, Bethesda, MD, USA). Lesion and hemisphere areas were manually traced on individual sections throughout the brain. Lesion volume was calculated by multiplying surface of all traced areas by the sum of the section thicknesses plus the intersection distances.

### Immunohistochemistry

For fluorescent immunohistochemistry, cryostat 35 µm-thick coronal sections (prepared from the same brains as above) were rinsed in PBS 4 times for 5 minutes in order to remove the O.C.T. compound. Sections were blocked for 30 minutes in PBS containing 10% goat serum and 0.25% Triton X-100. Incubation with anti-NeuN (Millipore, Billerica, MA, USA) primary antibody (mouse monoclonal, 1:300 dilution) was performed overnight at room temperature in PBS buffer containing 1% goat serum and 0.25% Triton X-100 (both Sigma Aldrich). Control slices were incubated overnight in a buffer that was not containing primary antibody. Slices were rinsed with PBS containing 0.25% Triton X-100 4 times for 5 minutes. Next, slices were incubated for 2 hours at room temperature in goat anti-mouse Alexa 488 secondary antibody (Invitrogen) at 1:500 dilution. After incubation, slides were washed with PBS containing TritonX-100, mounted with Fluoromount Aqueous Mounting Medium (Sigma Aldrich), coverslipped, and left overnight to dry. Brain sections were examined under a fluorescence microscope and photographed under 20 × magnification on both, ipsilateral (ischemic) and contralateral side.

### Quantification and statistical analysis

As previously described (17), ischemic lesion volumes were measured indirectly by subtracting the volume of nonischemic (healthy) tissue of the ipsilateral (ischemic) hemisphere from the total volume of contralateral (healthy) hemisphere. Brain edema approximation was expressed as percent increase of ischemic ipsilateral hemisphere volumetric size with respect to contralateral (non-ischemic) hemisphere (18). Since we isolated the brains 24 hours after MCAO, edema quantified here presented mainly vasogenic brain edema. All quantifications were performed by two independent “blinded” individuals in order to reduce bias. After quantification, linear regression analysis was performed in order to compare lesion volumes and brain edema obtained by microCT device with volumes measured on histological sections (GraphPad Prism 5.00a, GraphPad Software Inc., San Diego CA, USA).

## Results

**Figure 1:**
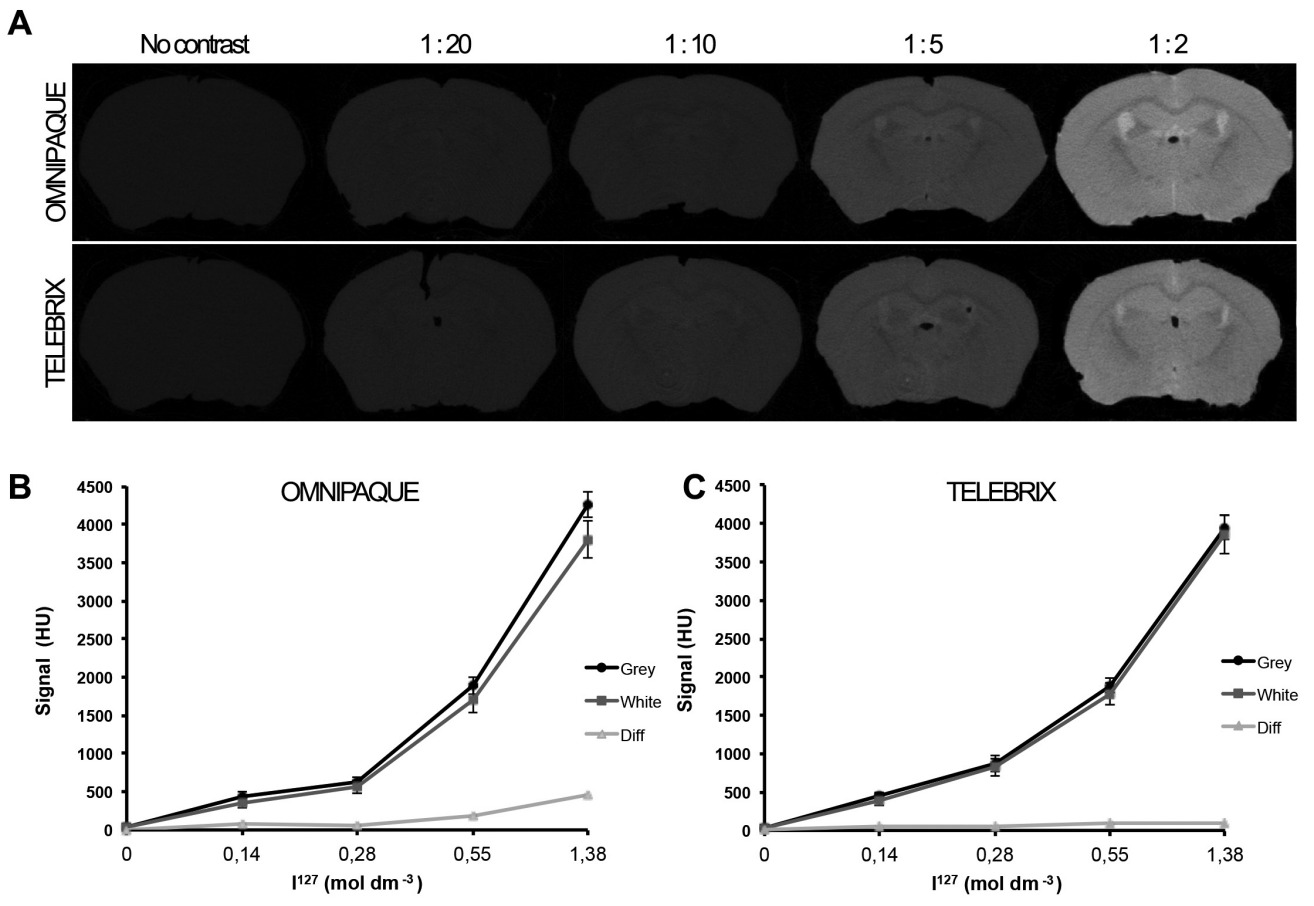
(**A**) Comparison of coronal sections from microCT acquisitions of mouse brains. Mouse brains were immersed in phosphate buffered saline (PBS) and in different dilutions (1:20 to 1:2) of two contrast agents Omnipaque (first row) and Telebrix (second row) in PBS. Signal intensity diagram expressed in Houndsfield units (HU) showed the different values of gray and white matter depending on the concentration of I^127^ ions in Omnipaque (**B**) and Telebrix (**C**) contrast agents. Difference between gray and white matter signal intensities was indicated as “Diff.” No native brain contrast was seen in pure PBS, while the best gray/white matter signal difference of 460 HU was noticed in the 1:2 dilution of Omnipaque contrast agent (**B**). (**C**) Telebrix contrast agent provided relatively poor gray to white contrast with gray/white matter signal difference of 89 HU. Data are expressed as mean ± standard deviation.

To select either non-ionic or ionic RCAs (Omnipaque and Telebrix) for *ex-vivo* brain imaging, we compared microCT acquisition signals of mouse brains immersed in contrast agents at different iodine concentrations. Brains immersed in PBS served as a negative control for contrasting and showed a weak signal, with no soft tissue contrast (Figure 1A). Application of two different RCAs resulted in different signal intensities between gray and white matter depending on the concentration of iodine ions (Figure 1A). Both, Omnipaque and Telebrix diluted 1:2 with PBS at the same iodine concentration showed the best gray/white matter contrast signal. For 1:2 dilution of Omnipaque, the gray/white matter signal difference was 460 HU, while for Telebrix it was 89 HU (Figure 1B and C). Since non-ionic RCA Omnipaque showed better gray/white matter contrast, allowing the appropriate visibility of morphological features of the brain, it was chosen for all further experiments.

**Figure 2:**
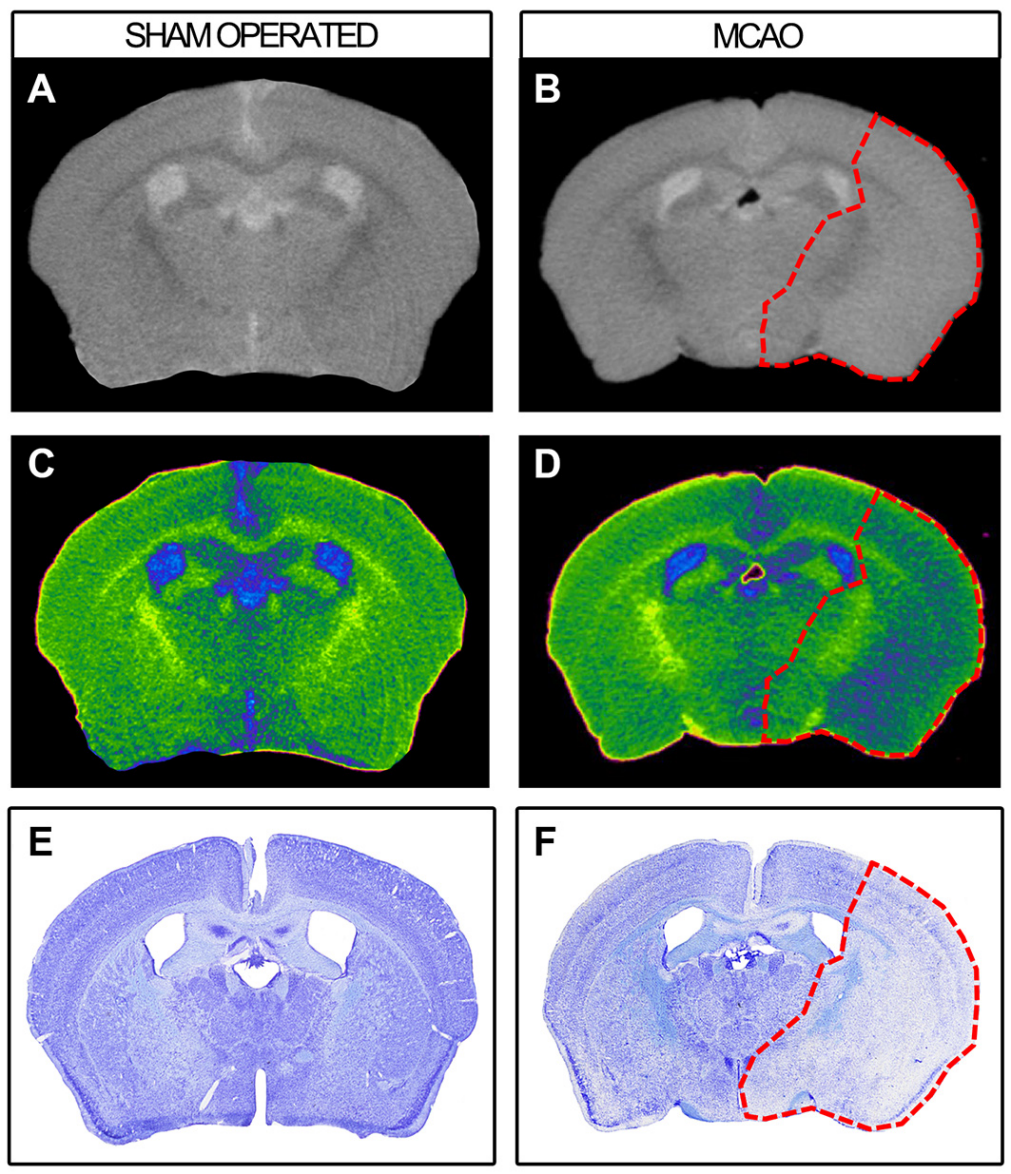
Comparison between corresponding coronal brain slices of the same brains obtained by microCT imaging, presented in raw format (**A, B**), color enhanced visualization (**C, D**), and Nissl staining (**E, F**). Left column of images presents sham-operated animals with no visible difference between the two hemispheres (**A, C, E**). Right column of images presents visualization of coronal brain sections after transient middle cerebral artery occlusion (**B, D, F**). Dashed red line separates the area of ischemic lesion from the surrounding healthy tissue.

In order to verify whether microCT is a valid method for visualization of brain ischemic lesions, we compared microCT images with appropriate Nissl stained brain sections of sham operated (Figure 2A, C, and E) and MCAO operated mice (Figure 2B, D, and F). In order to enhance visualization of minor morphological details on microCT images, we changed the color scale visualization from grayscale to color scale 1 available in the CTAn software, which facilitated the detection of the lesion area (Figure 2C and D). microCT images and Nissl stained sections of sham operated mice showed no difference in morphology between the two brain hemispheres (Figure 2A, C, and E). In contrast, images of lesioned brains revealed difference in morphology between ischemic and non-ischemic areas (Figure 2B, D, and F). The area of ischemic lesion was presented on microCT imaging as an area with higher signal intensity, with clear delineation of the ischemic area from the surrounding healthy tissue (Figure 2B and D). The line marked on microCT images was almost identical to the one marked on Nissl stained sections (Figure 2B, D, and F). These results showed that microCT imaging with the use of nonionic contrast agent (Omnipaque) served as a valuable method for detection and visualization of ischemic lesions, with similar sensitivity to the one of traditional methods such as Nissl staining (Figure 2D and F).

**Figure 3:**
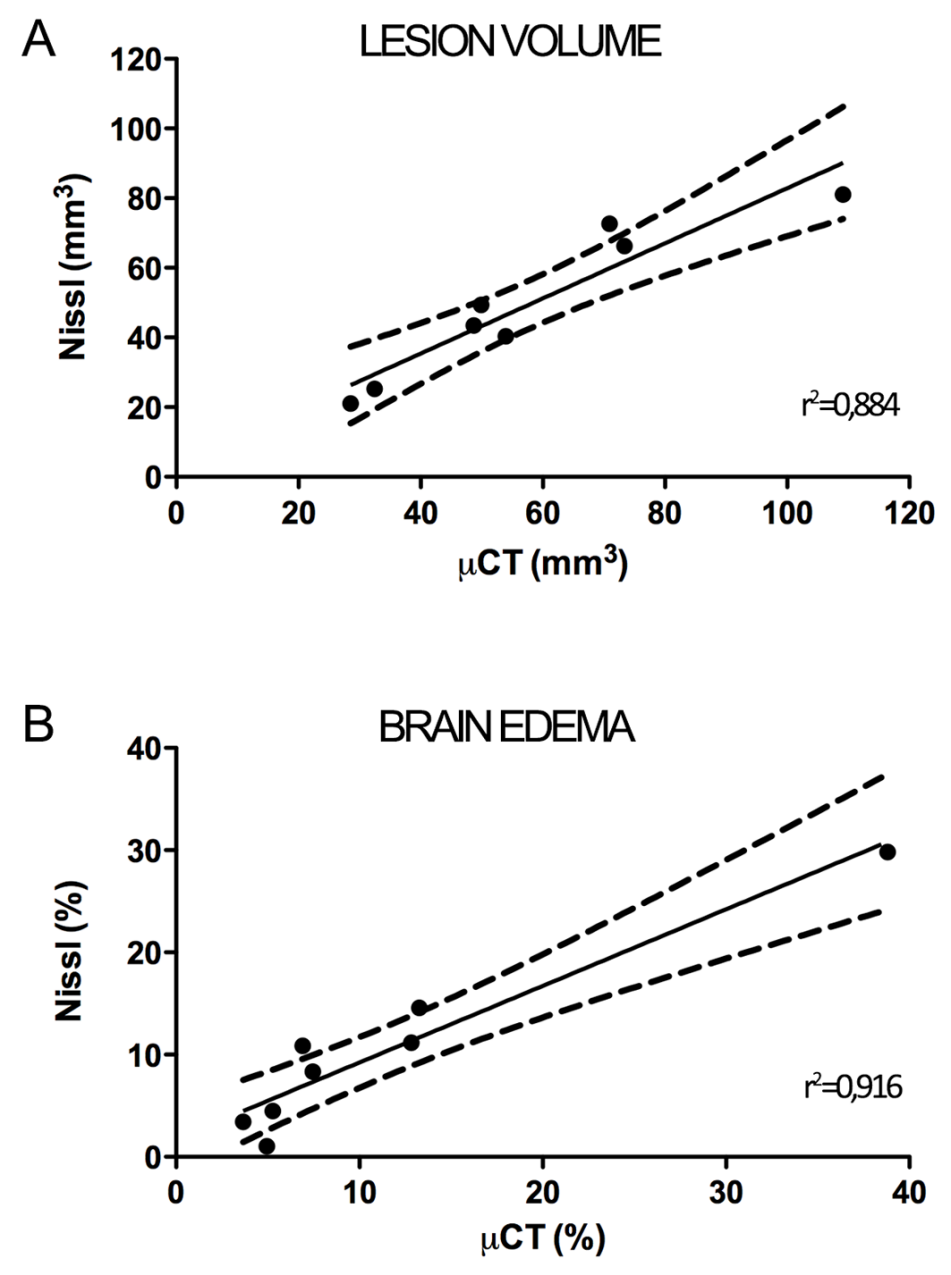
Linear regression analysis of microCT- and Nissl-stained lesion volumes (**A**) and brain edema (**B**) measurements. The x-axis represents microCT volumetric analysis; the y-axis shows corresponding data calculated from serial histological sections. Brain edema was measured as the percent of increase in hemisphere size of the right damaged hemisphere compared with left undamaged hemisphere. With the intercept fixed at zero, there was a significant correlation between the two techniques (ischemic lesion r^2^ = 0.884, *P* = 0.0005; brain edema r^2^ = 0.916, *P* = 0.0002).

To detect if the lesion volume quantification analysis performed by microCT imaging was comparable to those obtained by standard histological methods such as Nissl staining, we performed the two analyses, microCT and subsequently Nissl staining of serial histological sections on the same brains. The linear regression analysis of lesion volumes quantified by microCT and by Nissl stained sections showed a significant correlation between the two data sets (r^2^ = 0.884, *P* = 0.0005) (Figure 3A). The percent of brain edema (ie, increase in the hemisphere size of the ipsilateral [ischemic] hemisphere, quantified as percent of undamaged contralateral hemisphere) for the microCT and histology (Nissl stained) data sets also showed a significant correlation (r^2^ = 0.916, *P* = 0.0002) (Figure 3B). These results suggested that microCT was a valid method for ischemic lesion volume quantification and cerebral edema estimation.

**Figure 4:**
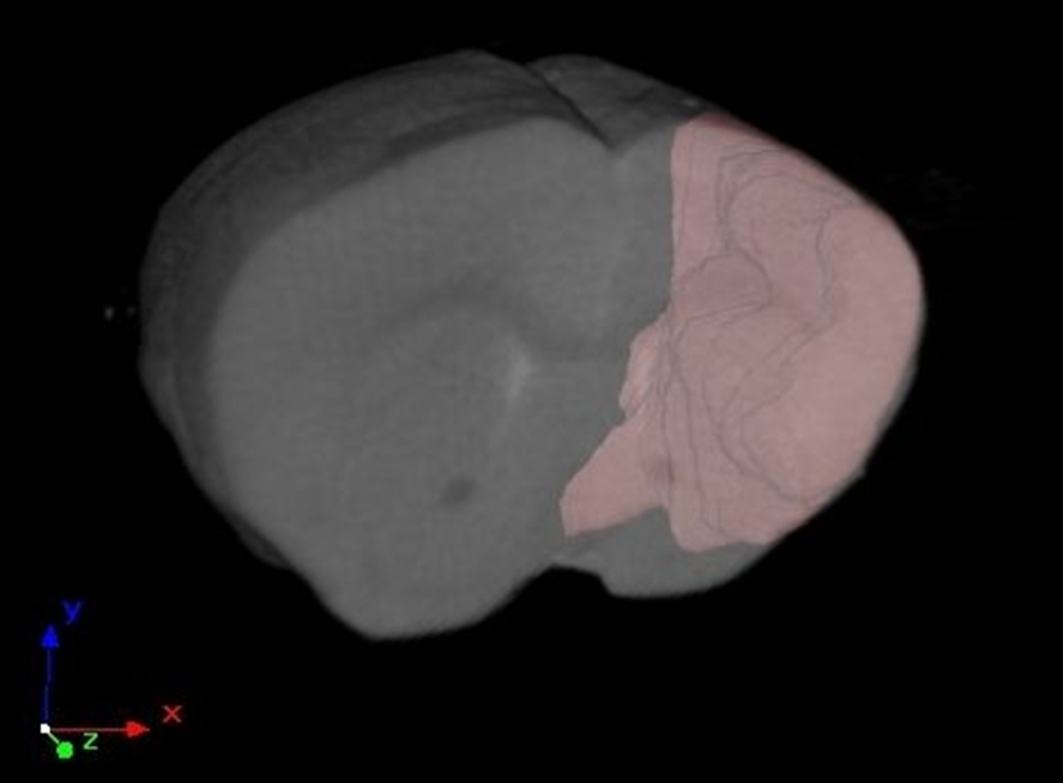
MicroCT allows high-resolution three-dimensional reconstruction of the entire mouse brain clearly delineating the lesion inside the ipsilateral infracted hemisphere.

Furthermore, in order to verify the microCT advantages over standard histological methods, a three-dimensional imaging of brains that underwent MCAO procedure was performed. MicroCT was proved to be a valuable tool for three-dimensional imaging, enabling high-resolution reconstruction of the entire mouse brain and volumetric analysis of the ischemic lesion inside the infarcted hemisphere (Figure 4, supplementary video)(web extra material 1).

**Figure 5:**
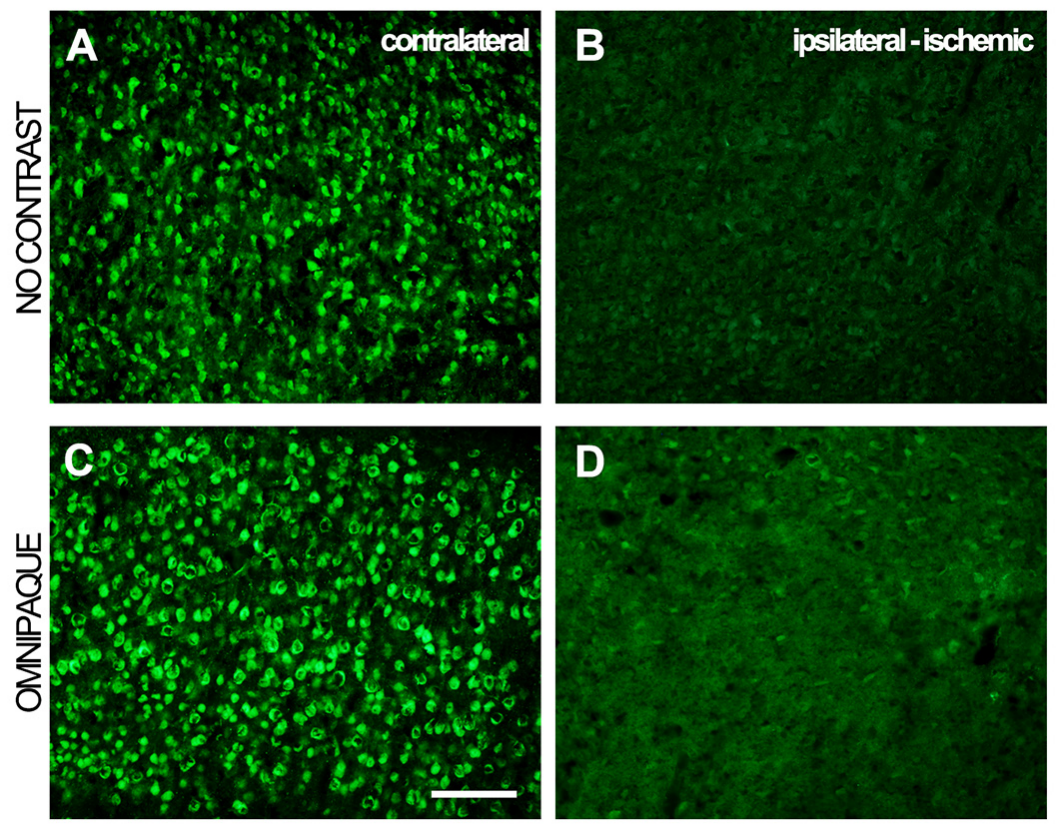
Coronal brain sections of the mouse that underwent middle cerebral artery occlusion (MCAO) procedure stained for neuronal nuclei (NeuN). Loss of NeuN immunoreactivity following ischemic injury compared to the contralateral side present on brain sections immersed in Omnipaque (**C, D**), compared to the control brain sections showing the same fluorescent signal following ischemic injury compared to the contralateral side (**A, B**). Scale bar, 100 µm.

In order to verify if brains immersed in Omnipaque RCA may be used for other investigations, such as immunofluorescent analyses, after microCT imaging we used antibody against neuronal nuclei, anti-NeuN, one of the most commonly used antibodies for the brain. The advantage of this antibody is that a loss of NeuN immunoreactivity occurs after onset of brain ischemia (19,20). A clear decrease in NeuN signal following ischemic injury (Figure 5A) compared to the contralateral side (Figure 5B), was observed. The same contrast in NeuN immunoreactivity between lesioned and unaffected brain tissue was also present before the immersion in Omnipaque RCA (Figure 5C and D), demonstrating that Omnipaque RCA did not affect the original tissue immunoreactivity.

## Discussion

In preclinical trials on animal models of stroke there is a constant need for an appropriate imaging method and quantification of lesion volumes in order to reveal even the minor therapeutical effects of potential neuroprotective drugs. The ideal tool for quantification and delineation of ischemic lesions should be rapid, noninvasive, and cost-effective, allowing direct measurement of ischemic lesion volumes. This study showed that microCT can be used in this context as a reliable tool for visualization and quantification of brain ischemic lesions in the mouse.

Previously reported x-ray contrasting methods highlighted the value of microCT for imaging rabbit and mouse brains, confirming the applicability of contrast stains for imaging of different soft tissues (6,12). In contrast to the previously reported use of ionic contrast agents, in our study the use of non-ionic Omnipaque contrast agent resulted in better-contrasted signal, providing appropriate morphological information. The use of contrast stains for the visualization of ischemic lesion was possible, because of the loss of gray/white matter borders and an overall increase in tissue water content, which reduced attenuation due to the brain edema after brain ischemia (21). Density changes on microCT images depended upon local densities of electrons and higher iodine concentrations in the infarcted lesion area (due to the formed edema). The suggested mechanisms enabled to delineate the ischemic lesion from the surrounding non-infarcted brain tissue.

The visualization of brain ischemic lesion by microCT was verified by Nissl staining of serial sections of the same brains. Nissl staining method offered the necessary precision to analyze the lesion, showing the metabolically compromised cellular elements as poorly stained (5,22). MicroCT enabled a high-resolution reconstruction of the entire mouse brain and volumetric analysis of both hemispheres. The results of microCT correlated significantly to those obtained by Nissl-staining method, with the advantage of microCT being that it avoids shrinkage during specimen preparation and complicated 3D reconstruction from section to section.

Quantification of cerebral edema is considered an important tool in assessing the stroke consequences (23). Various vasoactive compounds can modulate the extent of cerebral edema, and in that way, they may change the pathophysiological cascade following stroke and have either beneficial or detrimental effects on the recovery processes following ischemic lesion (24). In our study, we showed that microCT could be used for the assessment of cerebral edema, as verified by comparison to histological analysis using Nissl staining.

The immersion in RCA was necessary for microCT analysis; still the same ex vivo brain specimen was used for further immunohistochemistry investigations, not only for the classical method of Nissl staining. This is very important for reducing the number of animals used for the experiments. The future application of different antibodies should further test whether RCAs affect original tissue immunoreactivity.

Although the same conclusions could be drawn from microCT imaging and traditional histology, it is important to mention that microCT allowed measurement without destructing the specimens, allowing rapid identification and quantification of the ischemic lesion and reducing the time spent on histology. This all makes microCT a powerful tool for easy, rapid, noninvasive, and cost-effective imaging of the mouse brain ischemic lesions.
